# First Do No Harm: An Analysis of the Risk Aspects and Side Effects of Clinical Holistic Medicine Compared With Standard Psychiatric Biomedical Treatment

**DOI:** 10.1100/tsw.2007.268

**Published:** 2007-11-12

**Authors:** Søren Ventegodt, Isack Kandel, Joav Merrick

**Affiliations:** ^1^ Quality of Life Research Center, Teglgårdstræde 4-8, DK-1452 Copenhagen K, Denmark; ^2^ Research Clinic for Holistic Medicine, Copenhagen, Denmark; ^3^ Nordic School of Holistic Medicine, Copenhagen, Denmark; ^4^ Scandinavian Foundation for Holistic Medicine, Sandvika, Norway; ^5^ Interuniversity College, Graz, Austria; ^6^ Faculty of Social Sciences, Department of Behavioral Sciences, Ariel University Center, Samaria, Ariel, Israel; ^7^ National Institute of Child Health and Human Development, Jerusalem, Israel; ^8^ Office of the Medical Director, Division for Mental Retardation, Ministry of Social Affairs, Jerusalem, Israel; ^9^ Kentucky Children's Hospital, University of Kentucky, Lexington, KY, USA

**Keywords:** Clinical holistic medicine, mental health, alternative and complementary medicine, psychiatry, psychiatry

## Abstract

Clinical holistic medicine (CHM) is short-term psychodynamic psychotherapy (STPP) complemented with bodywork and philosophical exercises, to be more efficient in treating patients with severe mental and physical illness. STPP has already been found superior to psychiatric treatment as usual (TAU) and thus able to compete with psychiatric standard treatment as the treatment of choice for all non-organic mental illnesses; we have found the addition of bodywork and philosophy of life to STPP to accelerate the process of existential healing and recovery (salutogenesis). In this paper we compare the side effects, suicidal risk, problems from implanted memory and implanted philosophy of CHM with psychopharmacological treatment. Method: Qualitative and quantitative comparative review. Results: In all aspects of risks, harmfulness, and side effects, we have been considering, CHM was superior to the standard psychiatric treatment. The old principle of “first do no harm“ is well respected by CHM, but not always by standard psychiatry. CHM seems to be able to heal the patient, while psychopharmacological drugs can turn the patient into a chronic, mentally ill patient for life. Based on the available data CHM seems another alternative to patients with mental illness. There seem to be no documentation at all for CHM being dangerous, harmful, having side effects of putting patients at risk for suicide. As CHM uses spontaneous regression there is no danger for the patient developing psychosis as, according to some experts, has been seen with earlier intensive psychodynamic methods. CHM is an efficient, safe and affordable cure for a broad range of mental illnesses.

## INTRODUCTION

Integrated science and integrative medicine has become increasingly popular, both with scientists and with patients. The research in issues like scientific holistic medicine and quality of life has exploded the last decades, as a search on www.pubmed.gov will show. Out of the boiling pot of contemporary, integrative, medical science has several, quite different, new treatment systems crystallized. One of the most interesting new systems is clinical holistic medicine (CHM)[1-40] developed in an international network of medical and psychological researchers and tested at the Research Clinic for Holistic Medicine at the Quality of Life Research Center (NGO) in Denmark. CHM aims to integrate epidemiological research on quality of life and health[41], Hippocratic character Medicine[42,43,44], psychosocial medicine focusing on Antonovsky and sense of coherence[45,46], psychoanalysis and psychodynamic therapy (primarily short-term psychodynamic psychotherapy, STPP[47,48,49]), and finally transcultural medicine and CAM, both in theory, methodology and clinical practice (primarily bodywork addressing the patients unconsciousness and emotional layers, like the Marion Rosen Method[50]).

The most resent development has been an attempt to treat mentally ill patients with CHM, often psychiatric patients not responding to biomedicine and quite surprisingly it seems that this system is able to help most of the patients, without the suicidal risk and the other severe side effects of the traditional psychiatric biomedical treatment[35,39]. Unfortunately the documentation is still based on a limited number of mentally ill patients. Because of its offspring in quality of life research it is focusing on the subjective experience of being mentally ill, not on objective symptoms and diagnoses of specific psychiatric disorders. Most fortunately CHM is build on the strong traditions of clinical practice and research of psychodynamic psychotherapy (STPP) that is known to be almost completely free of side effects/adverse-effects as documented by a search on Medline (www.pubmed.gov). The search for "psychodynamic psychotherapy AND adverse effects" gave only 16 hits with none of them reporting adverse effects and some reporting no adverse-affects. A similar search for "psychodynamic psychotherapy AND side effects" gave 19 hits none reporting side effects and some reporting no side effects. A similar search for "drugs AND side effects" gave 138,726 hits, and "drugs AND adverse-effects" gave 128,059 hits, a large fraction of these with positive findings. Side effects and adverse-effects are obviously a problem connected to the use of drugs, and not to psychodynamic psychotherapy.

As the experience of being mentally ill is what basically torments the patients the most, the subjective improvement of this aspect might very well be the most important dimension of any psychiatric quality assessment and treatment-effect evaluation. More research should definitely be done to also document the objective aspects of the process of healing the mentally ill with CHM.

This paper is going to examine the most important differences between CHM and standard psychiatry regarding the risk aspects and side effects of the treatment.

## Standard Psychiatry

In biomedical psychiatry the theory is that mental illness is product of disturbed or deregulated brain chemistry. The cure is therefore drugs that regulate the brain activity in different neurotransmitter pathways. The reality is complex with many antipsychotic and antidepressive drugs acting on many different transmitter systems. Effective drugs are available for stimulating depressed patients, tranquilizing anxiety patients, and down regulating overactive, psychotic patients.

Unfortunately these drugs all have quite severe side effects, with a number needed to treat to harm (NNTH) around two and a number needed to treat to cure (NNTC) around four. Major side effects are excessive overweight, sedation, involuntary movements (dyskinesia), Parkinson's disease, suicide, and sudden, unexpected death. The drugs are in most cases not curative. When the patients stop taking the psychopharmacological drugs the original disease and the disturbed mental state of the patients is seldom bettered but quite often worsened. These facts make the standard psychiatric treatment a less than perfect treatment.

As many of the drugs are affordable and easy to use for the patient, in contrast to most relevant psychotherapy, and as mental illness are very common, these drugs have become extremely popular in most western countries. The strategy of treating symptoms with drugs and not curing the patients is creating a huge number of chronically ill patients, and the society at large is thus paying a huge prize in so many of its citizens not being able to function and work properly, and most mentally ill patients continue to suffering though their life, just at a diminished scale.

## Clinical Holistic Medicine

In scientific holistic medicine the theory is that mental illness is a product of disturbed or disregulated consciousness, causing disturbed brain neurophysiology, which again gives the behavior we observe as mental illness. The subjective experience of a dysfunctional or disturbed consciousness is the feeling of being mentally ill. The disturbance is due to compromised psychosocial and psychosexual development because of traumatic events and adaptation to a less-than-perfect childhood environment. These events has lead to negative learning about the world, “destructive decisions” in the language of CHM, an only by de-learning this, the normal mental functionality can be restored. The de-learning is happening though a process of healing that is facilitated by the combination of giving resources to the patient, and taking the patient back to the old wounds. The purpose of the treatment is to cure somatoform and psychoform dissociation[51,52], to enable the patient to once again be in full contact with the surrounding world though mind and body. This is also called the rehabilitation of the “sense of coherence”[53-57], or existential healing[58]. The sign of the healing taking place is first that the patient re-enters a number of historic life-crises with destructive learning, and then the sudden and dramatic improvement of self-assessed mental health, followed by rehabilitation of the patient’s ability of functioning. In the end the cure the patient knows himself and his major talents, and assumes responsibility to be an integrated and valuable part of the world. Thus CHM is curative, and helps the patient to get back to a normal function in society. The use of CHM takes a certain understanding of life and a certain level of willingness to suffer the old pains once again, and it can take years before a substantial betterment has occurred. About half of the mentally ill patients have been helped in one year and with 20 sessions of CHM, making CHM affordable for most patients[39]. The curative aspect means that all expenses to medicine come in the first years of treatment.

## NEGATIVE AND POSITIVE EFFECTS

## Physical – The Body

*Standard psychiatric pharmacological treatment:* The psychopharmacological drugs are known to be very hard to the body, with overweight, sedation, and involuntary movements and the most common, but hundreds of quite severe side effects are known, and sudden, unexpected death is happening 150% as often for patients using antipsychotic drugs[59].

> *CHM treatment:* As this system avoids the use of drugs, all the negative side effects of the drugs are not present in this system. The treatment is taking the patient back to old life crises, which is often very unpleasant, but as this is a part of treatment, and only lasting for hours or a few days, this is not considered a side effect. The most advanced tools of CHM like intensive bodywork on a floor with many holdings, taking the patient into re-experiencing birth (re-birth exercises[60]) has been seen to give the patients bruises and scratches but nothing that could not heal within a week. CHM has thus no known physical side effects.

## Physical – Future Health

*Standard psychiatric pharmacological treatment:* The future health of most mentally ill patients is pour, with a substantial loss of number of life years. The physical side effects tend to accumulate though the years, increasing the probability for the drugs being harmful.

> *CHM:* As the treatment addresses existence as a whole, subjective physical health is normally also improved, when subjective mental health is improved. CHM is effective as preventive medicine, and both physical, mental, sexual, and existential health problems seem to be prevented.

## Mental – The Mind

*Standard psychiatric pharmacological treatment:* The psychopharmacological treatment with psychopharmacological drugs are known to be very hard to the state of mind, with loss of motivation, interest, libido, sedation, lack of self-confidence and self-esteem, and suicidal thoughts and attempts[61]. Advanced studying is often not possible on these drugs[62], as they are known to give a restriction of the patient's learning capacity, which also may represent an obstacle to the application of other treatment modalities, e.g. behaviour modification, or to the patient's social re-adaptation. Thus the patients and the experiential life is often dramatically reduced[62]. The consequence of this is that the development of insight in self, others, and society often is arrested, and the patient remains though life at an infantile level of mental development.

> *CHM:* As this treatment is accelerated personal development, it develop self-insight and responsibility for own life, and increases dramatically the experiential life and the reflections upon the experiences. The use of talents is often dramatically improved, and training the patient in being of value to the surrounding world is a standard part of CHM treatment. The ability to go though advances studies, like university studies, are often dramatically increased. As CHM uses spontaneous regression, where the patient returns to the trauma in surplus of resources, the therapeutic process cannot get stuck, and there is therefore no danger for the patient developing psychosis, as has been seen with earlier, intensive psychodynamic regression methods.

## Mental – The Mental Future of The Patient

*Standard psychiatric pharmacological treatment:* The most important negative statistically documented consequence of using psychopharmacological drugs is chronicity – becoming a chronic patient. The reason for this is arresting personal development and the drugs directly and indirectly hindering existential healing and recovery.

Another serous problem is the psychopharmacological drugs psychodynamic interference with psychotherapy, e.g. by diminishing the patient's motivation to pursue this type of treatment or by disturbing the structure of his defences[62].

An important problem arising from the implanted philosophy of psychiatric treatment is what could be called “the trap problem”: If a patient chooses to enter psychiatric treatment, and gets the diagnosis of schizophrenia, and is having the antipsychotic drug treatment, the philosophical impact of this treatment is often so dramatic that the patient is trapped in his own understanding of himself as a chronic mentally ill patient with no future socially or work-wise, and in this resignation all motivation for helping himself to healing and recovery is lost. The trap-problem is mirrored in the finding that there exist almost no successful studies of hospitalized patients being cured by psychodynamic psychotherapy[63], while there are many such reports independently of the psychiatric treatment institution[64]. Because of the faith-determining step of psychiatric hospitalization the conclusion that schizophrenia cannot be cured with psychodynamic psychotherapy is not likely to be correct, and we have seen schizophrenia patients seemingly being cured with CHM.

> *CHM:* With the development of sense of coherence and increased responsibility and self-insight, the mental future for the CHM patients healing successfully in therapy is bright. They will most often be an integrated an active part of society though their life, and they will fight for what they believe, because of the level of self-consciousness and self-esteem they develop in the therapy.

## Sexual

*Standard psychiatric pharmacological treatment:* The drugs are known to be very hard on the libido as well as on the general motivation[62] and mood[65]. Lack of desire and sexual interest is normal with the mentally ill treated with antipsychotic and antidepressant drugs. In the male, a common problem seems to be diminished potency; as this has not been thoroughly investigated more research is needed on the side effects on sexuality.

> *CHM:* rehabilitation of sexuality of a standard element of the CHM treatment, as psychosexual health is seen as closely connected to mental health. Therefore the sexual life of CHM patients is most often radically improved[36,37,39].

## Behavioral

*Standard psychiatric pharmacological treatment:* The behavior of the patients is often quite bizarre, with many involuntary movements, and a most peculiar movement pattern easy to detect for a normal person, which often leads to severer marginalization of the mentally ill patients on psychopharmacological drugs. As years goes by, the arrested psychosocial and psychosexual development from the reduced experiential life, will also lead to a behavior that is very characteristic and often seen as infantile. The sedation, overweight and other side effects often also impacts the behavior negatively, giving the patient on psychopharmacological drugs a severe disadvantage compared to the normal population.

> *CHM:* The accelerated personal development and increased self-insight from cultivated reflection is often making the behavior of the patient more elegant and attractive. In general the CHM patients are doing very well socially, work wise, sexually etc.[35-39]. The negative side effects seen with drugs are not present here.

## Financial

*Standard psychiatric pharmacological treatment:* As most mentally ill patients using psychopharmacological drugs are not able to compete with normal individuals, their ability to get and function in a job is often compromised. This leads to a severely reduced personal income; often the patients will end up with a social pension.

> *CHM:* As the mentally ill patients not only is cured but also taking into accelerated personal development, witch often will continue after therapy, the ability to work will often increase radically, giving the patient not only a normal ability to work, but often allowing the cured patient to study at university and rehabilitate the use of core talents, to the benefit of themselves and their surrounding world.

The cost of treating a patient with STPP or CHM is between 1-4000 Euros; the standard psychiatric treatment that reduces the patients to chronic mentally ill patients for life with frequent hospitalization is often over 1 mill. Euros[see 64 for some calculations].

## Social

*Standard psychiatric pharmacological treatment:* All the above-mentioned aspects sums up to a severe social handicap for mentally ill patents using the psychopharmacological drugs.

> *CHM:* All the above mentioned aspects sums up to a severe social empowerment for the mentally ill patients choosing accelerated personal development as way out of the mental illness.

## Quality of Life

*Standard psychiatric pharmacological treatment:* In Denmark this is the standard treatment for mental illness, and the mentally ill is known to have a very low QOL in Denmark. Ventegodt found the happiness of these patients to be 21.4% under average, and the IQOL to be 21.8% under. The use of drugs to the central nervous system is also directly associated with a very poor QOL; the happiness for the patients using this was 19.7% below average of the Danish population[62,63].

> *CHM:* CHM is known to improve QOL, as it was originally designed exactly to do this. Ventegodt et al. found a very strong association between self-asses mental health and QOL in nine different dimensions, signifying the importance for QOL of improving subjective mental health[63].

## Spiritual

*Standard psychiatric pharmacological treatment:* The spiritual well-being is in science most often connected with the experience to be an integrated and happy part of the world; the concept of “sense of coherence” developed by Antonovsky and further by Lindström et al[64], Flensborg-Madsen et al.[52-57], and Ventegodt et al.[44], is strongly connected to subjective mental health, and quality of life. As neither global quality of life nor subjective mental health seems to be normalized with psychopharmacological drugs in general, we must assume that SOC is not reestablished either by this treatment.

> *CHM:* Improvement of SOC is the core ambition in CHM; this happens in the process of existential healing described in[58]. The common patent's experience of this treatment is that spiritual well-being is radically improved; the meaning of life is appearing, the libido and reason to be is enhanced, and the being in general is consolidated. CHM makes the patient a happy and uncomplicated part of the world.

## Implanted Philosophy, Implanted Memory, and Suicide

*Standard psychiatric pharmacological treatment:* In this system the patients are thought the psychiatrists belief about their mental illness. Most often they learn that their disease is inborn, presumably a consequence of some dysfunctional or mutated genes (DNA) causing disturbances in the brains chemistry, and therefore giving the patient her symptoms; The patient will learn that the illness is incurable but that the drugs are likely to alleviate many of the symptoms of the disease. The side effects of the drugs are a reasonable prize, and most be tolerated.

This learned view of self as chronically ill is often putting the patient into deep existential trouble: why am I here, if I am no good? What is the meaning of my life, if I am to be a burden to the world for life? Why am I so unlucky that I got the bad genes so I cannot improve? The understanding that the patient is chronically ill and must lean all his hope for cure and comfort onto the doctor, is severely disempowering him, and often leading to severely diminished self-esteem and self-confidence. The suicidal thoughts are a logical consequence of this implanted philosophy. When the patient re-interpret his whole personal history, he will be more likely to blame the "genes" for all the things that went wrong for him, putting his own responsibility aside, hindering him learning from his past. This is "implanting memories", in the most general and destructive meaning of the concept.

The tremendous raise of suicidal attempts and suicides after the patients enter the standard psychiatric treatment is showing that the problems of psychological side effects of the psychopharmacological drugs and of implanted philosophy by the treatment has not been solved by the psychiatrists[61]. It has been suggested that the many suicides and suicide attempts is a direct consequence of the well-known dysphoria often induced by the psychopharmacological drugs[see 65 for a review].

> *CHM:* The CHM treatment is often extremely intense emotionally, and it is implanting the CHM philosophy of personal development of life, existence and consciousness, into the patient: "Everything that happens is in the end your responsibility, as you are free to perceive the world as you please. The gab you see is your responsibility, so please go and make your life good as your want it. Every defeat and failure is an occasion to take learning, and the fundamental reason to be here is to be so intimately connected to the world that you can use your talents to create value for your self, your loved ones, and your surrounding world".

Very often symbolic traumas of sexual nature is encountered in CHM therapy, rehabilitation also the libido of the patient, and quite often these traumas are taken for real in a phase of the therapy, but in the end they will be correctly perceived as the CHM therapy is ingeniously designed to help the patient to de-learn the implanted philosophy an to conquer true independence of other people, also the therapist himself, in the end of the therapy. Therefore the CHM is not implanting philosophy of memory in the patient, unless the CHM treatment for some reason is disrupted, which is most unfortunate; no severe harm to the patient has seemingly be done to any patient, even in this case. Suicide or suicide attempts provoked by CHM has not been observed in spite of many hundred patients now treated with this new method.

### Ethics – To Heal The Patient, Alleviate Human Suffering, and Do No Harm

*Standard psychiatric pharmacological treatment:* what has been characterizing psychiatry through the last century is extensive use of force. The problem is that use of force almost always is harmful and giving trauma. And in a way a sedating drug is also a forceful intervention in itself. The behavior of the psychiatric patient has often been severely impacted by the collection of trauma from the forceful psychiatric treatment itself. The classical "madman" almost exploding with rage just from a minute provocation is the typical consequences of massive use of force in psychiatry. The sad fact that QOL and subjective mental health is not alleviated in most cases by the use of psychopharmacological drugs, while the negative side effect and negative consequences of implanted memories are massive, like suicide and suicide attempt, is making the standard psychiatric treatment a less than optimal treatment. The fine Hippocratic principle of "First do no harm"[42] seems therefore not well respected by standard psychiatry.

A more complicated, ethical aspect of psychiatric standard treatment is the objectification and alienation of the patient that is a consequence of the objective analysis made of the patient in this system.

Another problem is the reduction of the physicians' therapeutic efficacy if he relies exclusively on psychotropic agents[62].

> *CHM:* "First do no harm" is a principle well respected by CHM. The use of force is normally very moderate or absent. The patient is seen and met as a living, autonomous, responsible, and consciousness subject. The existential healing is improving all aspects of patient's life, and is giving the patient a huge empowerment, by accelerating his personal development and self-insight. The patient is likely to be cured and thereafter lead a life of high quality, being happy and of value to himself, his family and friends, working place, and surrounding world at large. As conscious being he is likely to take good care of his world, from his son and loved one to the global ecosystem.

## CONCLUSIONS

In all aspects of risks, harmfulness, and side effects, we have been considering, CHM is superior to the standard psychiatric treatment. The old principle of "first do no harm" is well respected by CHM and seems to be able to heal the patient, while psychopharmacological drugs can turn the patient into a chronic, mentally ill patient for life. Based on the available data CHM seems to be an alternative choice regarding patients with mental illness. There seem to be no documentation at all for CHM being dangerous, harmful, having side effects of putting patients at risk for suicide. As CHM use spontaneous regression there is no danger for the patient developing psychosis as, according to some expert's opinions, in single cases has been provoked by earlier intensive psychodynamic methods; we find it more likely that these episodes were what we today would call "developmental crisis"[32] and not real psychotic episodes.. As the documentation still only includes a very limited number of patients treated with CHM, more research is needed to allow for stronger conclusions related to the specific psychiatric diseases, especially schizophrenia where the treatment with drugs like Chlorpromazine has shown itself to be particularly problematic[69].

Clinical holistic medicine (CHM) is based on short-term psychodynamic psychotherapy (STPP) that has a well-documented effect on the specific psychiatric disorders[47,48,49]; CHM seems to intensify and accelerate therapy so we have no reason to believe that CHM is less effective than STPP. CHM is an efficient, safe and affordable cure for a broad range of mental illnesses.
